# Online Group Music-Making in Community Concert Bands: Perspectives From Conductors and Older Amateur Musicians

**DOI:** 10.3389/fpsyg.2022.878307

**Published:** 2022-06-30

**Authors:** Audrey-Kristel Barbeau, Mariane Generale, Andrea Creech

**Affiliations:** ^1^Département de Musique, Faculté des Arts, Université du Québec à Montréal, Montreal, QC, Canada; ^2^Schulich School of Music, McGill University, Montreal, QC, Canada

**Keywords:** group music-making, community music, older amateur musicians, pandemic (COVID-19), online music

## Abstract

At the beginning of the pandemic, many music ensembles had to stop their activities due to the confinement. While some found creative ways to start making music again with the help of technologies, the transition from “real” rehearsals to “online” rehearsals was challenging, especially among older amateur musicians. The aim of this case study was to examine the effects of this transition on three community band conductors and three older amateur musicians. Specific objectives were to explore (1) intergenerational relationships to support online group music-making; (2) digital literacy and access in later life; and (3) online music-making in a COVID-19 context. Semi-structured interviews were conducted and theoretical thematic analysis was undertaken (Braun and Clarke, 2006). Results were analyzed from the conductors’ and older musicians’ perspectives, and common trends were combined to facilitate interpretation. The first theme showed that being part of an intergenerational ensemble contributed positively to the learning experience online. The second theme demonstrated that because both conductors and musicians were new to the online rehearsals, it contributed to attenuate the age-related digital divide that may have been observed in other studies. Regarding access in later-life, older musicians reported benefits associated with rehearsing online, specifically in terms of distance/commute, time, energy, and cost. However, for those who did not already have internet and electronic devices, the cost of acquiring all the necessary equipment to make music online could have been too high. Finally, the third theme revealed that musicians appreciated the opportunity to make music online and indicated that it was definitely better than having nothing, especially for its social aspects. In conclusion, while participants noted several challenges associated with online music-making (e.g., zoom fatigue and technological issues), they were also appreciative of the opportunity to continue making music at a time when in-person rehearsals were not possible. Pedagogical implications are discussed, specifically the importance of the support network, of meeting people where they are, of learning to adapt, and of collaborative teaching.

## Introduction

In March 2020, owing to the onset of the global COVID-19 pandemic, many music ensembles had to stop their in-person activities (Camlin and Lisboa, 2021, p. 129). Despite tumultuous circumstances, some found creative ways to start making music again with the help of digital technology. That said, the transition from in-person rehearsals to virtual ones was not an easy step, especially among older amateur musicians.

There exists a well-documented generational divide regarding the use of technology, with older adults being less likely to engage with technology than what is observed among younger generations (Charness and Boot, 2009, p. 253; Poushter, 2016, p. 6–23). That said, one study has shown that older adults are more compelled to engage with new forms of technologies when the perceived benefits outweigh the challenges associated with their technical use (Heinz, 2013, p. 69–70). In other words, despite feelings of frustration or inadequacy, older adults may be willing to learn to use new technologies if they perceive that these may be useful and contribute to their independence or quality of life (QoL). Additionally, for many older adults, music is suggested to be a significant resource in supporting their QoL, with some studies reporting benefits associated with the social and collaborative nature of group music-making (Coffman, 2002, p. 85; Creech et al., 2013, p. 96–98; Varvarigou et al., 2012, p. 183). Therefore, within the context of the pandemic, it became clear that to continue having access to this resource, one had to learn to make music within an online space (Camlin and Lisboa, 2021, p. 129). However, particularly during the earlier months of the pandemic, overcoming one’s fears or reservations about technology became a necessity in order to maintain participation and access to these group music-making activities.

For musicians whose music-making centered on chamber ensembles, wind bands, orchestras or choirs, continuing to pursue musical goals within those group contexts required a willingness to transition from practicing music weekly with others in a place-based rehearsal space to participating virtually within an online space. While the changes and restrictions introduced by the pandemic affected all musicians, as they limited (or even totally prevented) in-person collaborations and synchronous music-making (Fram et al., 2021, p. 2; Rosset et al., 2021, p. 9–10), these shifts came with multiple challenges and barriers that may have particularly affected older musicians (regardless of level). Examples include technological challenges (i.e., highly complex technology), technical challenges (i.e., unstable internet connection), and musical challenges (i.e., not being able to hear bandmates while playing). In an earlier study, Vaportzis et al. (2017, p. 1) had identified barriers to using technology among older adults, particularly when interacting with tablets. The authors concluded that most participants “were eager to adopt new technology and willing to learn” (2017, p. 1) but voiced some concerns about specific barriers (for instance, cost, lack of support or knowledge, feelings of inadequacy).

During the pandemic, older musicians were not alone in their struggle: all generations were forced to adapt to the new reality of COVID-19, from students and music teachers in elementary and high schools (Cheng and Lam, 2021; Boucher et al., 2022) to amateur musicians in community music schools (Salvador et al., 2021) as well as professional musicians (Coletto, 2020). As a result, musicians—regardless of age or background—pulled together to ensure that music would prevail during these trying times. That said, many music teachers felt they were not adequately prepared to make the shift, which generated stress and anxiety (Cheng and Lam, 2021, p. 211). The authors reported that music teachers were concerned about issues such as technological integration and the ability of students to adapt to online teaching. Fortunately, as Thornton (2020) reflected, there were ways in which the pandemic influenced music teachers and artists to respond to these new challenges, translating to an increase in creativity, a stronger sense of community and a responsiveness to students’ needs through empathy, patience and thoughtfulness. One particular example comes from Rowan (2021), who reported trying multiple methods (e.g., Skype, email, and Jamulus) in order to continue to provide music despite the fact that everything was online, illustrating the resiliency of conductors in such a challenging context.

The aim of this research was to examine the effects, in a pandemic context, of transitioning band rehearsals from in-person to an online environment on community band conductors and older amateur musicians. Specific objectives were to explore :

• Intergenerational relationships to support online group music-making.
• Digital literacy and access in later life.
• Online music-making in a COVID-19 context.

## Methods

### Design

This was a qualitative intrinsic case study (Creswell and Poth, 2018), bounded by the unique and unexpected circumstance that arose from the pandemic, whereby an adult community music activity (MNHB) had to transfer from an in-person to an online environment.

### Participants

This research project was approved by the ethics board of the Université du Québec à Montréal and the informed consent of participants was acquired by email prior to the start of the data collection process. Musicians were recruited within the Montreal New Horizons Band (MNHB), a bilingual and intergenerational community concert band for amateur musicians. Founded in 2014, the MNHB is a non-profit organization composed of three groups that are based on levels of musical proficiency: Initiation (for people who never played music before), Adagio (for beginner/intermediate musicians) and Allegro (for intermediate/advanced musicians). Three main conductors are usually involved with the organization (one for each group). Team-teaching is common practice among the conductors as all groups meet on the same evening (for instance, the main conductor of the Initiation band also acts as assistant conductor in the Allegro band). When not on the podium, all conductors play with the ensemble to support musicians. Music specialists are also hired as needed to lead sectionals. In addition, guest conductors are frequently invited (mostly students in Music Education who are following conducting classes at McGill University).

Before the pandemic, 86 musicians were registered to at least one group in the MNHB. In March 2020, all activities were suspended. Starting in Fall 2020, online activities were offered to Adagio and Allegro members. No Initiation band was offered as it was deemed too complex to introduce music to novices playing multiple (and different) wind instruments within an online context.

Online activities included two virtual bands (Adagio and Allegro levels, rehearsing weekly for 60–75 min between September and June), chamber music coaching (small ensembles rehearsing with a private coach for six 60-min sessions) and jazz workshops (a 10-week interactive course offered virtually between February and May 2021). All activities were provided by the regular MNHB conductors through Zoom Video Communications Inc (2022). Some of these conductors also offered private instruction online.

In the Fall semester, 20 musicians registered to one or more activities online. In the winter semester, 23 musicians registered and in the spring semester, 21 musicians. Table 1 describes the number of musicians registered in the virtual bands and chamber music coaching each semester as well as the number of people who abandoned their activities during the year. Participants in the jazz workshops are not included in this table due to the fact that the activity was offered for free to all members registered in one or more online activities. Therefore, the attendance was not taken and as a result, the attrition rate could not be reported (data are missing).

**TABLE 1:** Registration and attrition per online semester.

	Fall 2020		Winter 2021		Spring 2021	
	Registration	Attrition	Registration	Attrition	Registration	Attrition
Adagio	12	3	15	5	9^a^	1
Allegro	15	0	21	3	17^a^	0
Chamber music coaching	7	Not available	16	1	6^b^	0

*^a^These musicians continued on from Winter 2021.*

*^b^Participants rehearsed together outside following social distancing measures.*

It is worth noting that participation across two or three semesters was more stable for members of the Allegro band than the Adagio band. Some reasons for dropping rehearsals included health concerns, time conflicts with work, perceived difficulty of participating, and not appreciating the online format for rehearsals.

To recruit MNHB participants for the study, a convenience sampling method was used. Participants consisted of all the conductors teaching online in 2020–2021 (three female adults aged under 30 years) and three amateur musicians having participated in at least one online activity throughout the 2020–2021 season.

Conductors A and B were in charge of the virtual bands (intermediate and advanced levels). Conductor B was also responsible for all the online chamber music ensembles. Conductor C provided the jazz workshops for members. Conductor B and C also offered individual instrumental lessons online. All of them had completed at least a Bachelor’s of Music in their primary instrument and had taken undergraduate-level conducting classes sometime prior to the pandemic. Conductor A also had a Master’s in sound recording and was a Ph.D. candidate (Music Education) at the time of the study. Conductor C had a Master’s in instrumental conducting and was a music teacher in schools. Conductor B owned a music studio and was a full-time music instructor. They have been teaching and conducting within the MNHB for at least 4 years.

The musicians (two females and one male, aged between 67 and 79 years old) have been part of the MNHB for 5–7 years and were considered to be at intermediate levels on their main instruments. Additional demographic information appears on Table 2.

**TABLE 2:** Demographic information of study participants.

	Age as of Summer 2021 (years)	Involvement in MNHB (years)	Years spent playing primary instrument/conducting	Participation in other online musical activities
Conductor A	28	6	7	Yes^d^
Conductor B	28	7	7	Yes^e^
Conductor C	27	4	6	Yes^e^
Musician A	67	7	8^a,b^	No
Musician B	79	5	4^a,c^	Yes
Musician C	72	7	8^a,b^	Yes^e^

*^a^Took private lessons on their main instrument before joining MNHB.*

*^b^Novice: Started learning music later in life.*

*^c^Had played a variety of instruments before throughout their life.*

*^d^Took online private music lessons.*

*^e^Taught online private music lessons.*

It is important to mention that Conductor A, once the interviews were completed, joined the research team to contribute to the data analysis. While she was unaware (during the time she was conducting one of the virtual bands) that the transition from in-person rehearsals to virtual ones would become a specific focus for research within this context, her contribution to the analysis provided a valuable insider perspective and significantly enriched the credibility and trustworthiness of the analysis.

### Virtual Band Structure of Rehearsals

The virtual bands’ rehearsals were recorded and made available to ensemble members during the semester. Permission to record was asked at the beginning of each semester and participants who felt uncomfortable being recorded were suggested to keep their camera off. Unlike regular in-person rehearsals, the majority of the online band rehearsals were held in a sectional format, with musicians being split into their instrument families (woodwinds; Brass and percussion). Full band rehearsals were held three times during the semester—one at the beginning, one approximately in the middle, and one at the end of the term. Typical rehearsals usually started with announcements (if needed) or a question period, which was followed by a brief warm-up done with the full band. While most of the time all musicians had to be muted to adequately hear the conductor’s sound source, for warm-ups and slower chorale-like pieces, it was possible to have everyone unmute and play together. The ensemble was then separated into break-out rooms for sectionals or stayed together during full band rehearsals to work on pieces. Repertoire chosen for online music instruction were mostly flex band^1^ arrangements to allow more freedom regarding parts assignments. The rehearsal ended with a run-through of a piece (time permitting) or a question period. During rehearsals, musicians played along with either the conductor on keyboards or with recordings of the repertoire. Recordings were manipulated to be slower tempi using AudioTrimmer. Occasionally, musicians also received music theory and music history lessons during rehearsals. Both bands concluded their semesters with members individually recording themselves playing a selected piece to be put together for a “virtual concert” video (for more details on rehearsal structure, please refer to Supplementary Appendix 1).

### Data Collection and Analysis

Semi-structured interviews were conducted in French or English, based on the mother tongue of participants, and lasted approximately 45 min. All interviews occurred in July 2021, during summer break, after the end of all online activities. Interviews were transcribed in their original language. French quotes that were selected for analysis were translated to facilitate interpretation using DeepL, checked for clarity by a researcher fluent in French, and validated by the research team. Quotes that were selected to be included in the analysis were then sent back to each participant individually, to ensure that the written transcriptions were a representative depiction of his/her thoughts and experience.

The format of the interview consisted of 13 open-ended questions (Supplementary Appendix 2) that addressed three themes: (1) intergenerational relationships, (2) digital literacy and access in later-life, and (3) online music-making in a pandemic context. The intent was to seek participants’ perspectives in relation to these themes, with questions such as: “You participated in a virtual ensemble that was intergenerational. Do you feel that this intergenerational context may have improved your learning experience? If so, how?” (Theme 1); “How can virtual music-making maximize equitable access in later-life to creative musical expression, engagement in learning, digital literacy and quality of life amongst older citizens?” (Theme 2); or “What implications has the shift to an online environment had for you as a musician (or teacher)? What new skills or resources have you had to develop, in response to opportunities or challenges raised by the pandemic?” (Theme 3).

Following the guidelines set out by Braun and Clarke (2006, p. 83–85), theoretical thematic analysis was undertaken, meaning that data analysis was based on the researchers’ specific areas of interest and focused on specific aspects of the data rather than the entire dataset. Analysis was undertaken primarily at the “semantic” (or explicit) level, establishing patterns between extracts, and then attempting to understand their significance and establish their broader meanings in an iterative process. A research assistant, in charge of coding the data, was supervised by the main researcher through the first stage of analysis. Afterward, a second researcher checked the coding process and contributed to its improvement. Codes were reorganized as needed, based on a deductive approach. Participants’ perceptions of transitioning from in-person rehearsals to online rehearsals was examined from two perspectives: those of the teachers (adult band conductors) and those of the students (older adult band members). Results were combined to facilitate interpretation when trends were observed.

## Results

### Theme 1: Intergenerational Relationships to Support Online Group Music-Making

#### Intergenerational Interactions

The first question participants answered was associated with how they thought the intergenerational context of the virtual ensemble (namely, having teachers that were younger than them) contributed to their learning experience. From the band members’ standpoint, two main perspectives appeared—one in which the intergenerational relationship was helpful and one in which it did not have an impact on the learning experience. None indicated that having younger conductors was detrimental for their learning experience.

Among the participants who considered that the intergenerational context contributed to the learning experience, one musician thought about intergenerationality in relation to technology and problem-solving. The fact that people came from multiple technological backgrounds meant that there was a great deal of collaboration between participants:

[When there were] things that were not clear, there were always people in the group who were a little more competent in technology than others and then we don’t all get stuck in the same place. The things that I find super easy, others will find difficult and vice versa. So, the fact of being intergenerational, with technology evolving at a crazy speed, I think it’s really an advantage (Musician A).

For others, intergenerationality was not necessarily relevant to participants’ experience. For example, a musician indicated that they did not really focus on the intergenerational aspect when rehearsing, saying “to be honest, when you’re all together, you don’t see the intergeneration[ality] for all the group” (Musician C).

From the teacher’s standpoint, one conductor explained during the interview that being younger and still experiencing technological issues contributed to destigmatizing problems: “I think it helps that it was intergenerational ‘cause they saw that [Conductor B] and I would mess up sometimes too and if we could laugh about it, so could they” (Conductor A).

#### Explorations

Conductors and musicians were pushed to undergo exploratory phases at the beginning of the semester in order to problem-solve effective ways in which to do community band online. When asked to reflect on this, participants reported elements that worked particularly well and elements that did not.

Positive comments were made about the fact that commuting to rehearsals was not an issue anymore: Participants appreciated that tasks such as driving or commuting to and from rehearsals and carrying large instruments were absent for online rehearsals. Additionally, within the Montreal New Horizons Band, participants wishing for more playing time on their instruments had the opportunity to participate in the two bands that rehearsed back-to-back on Tuesday evenings (one intermediate band and one advanced band). With the virtual band, people kept this opportunity not only to play with both bands, but also to play different instruments in each band (for instance, alto saxophone in one, and tenor saxophone in the other). They could even switch instruments between pieces during the same band rehearsal. Therefore, because they did not have to physically carry multiple instruments to rehearsal, there was no space issue to keep both instruments beside them, and they had time to switch between one and the other as needed, they considered the experience as a positive. The online environment also offered a safe space to make mistakes, as no one (including the conductors) could hear when they made an error: “When we play, if our playing is not good or we’re not doing the right scale, there’s nobody who knows! [laugh]” (Musician A). In addition, some challenges associated with in-person playing were alleviated in the context of online music-making. For instance, Musician A explained that “for all the little 5 feet people, to be able to see the teacher who is conducting, when she is conducting and I have my tablet, I will say that it’s 50% of my pleasure.” She continued on to explain that while it was a big disadvantage to be small in life in general, it was worse in an orchestra because one constantly had to move to the right or left to see the conductor, and there is a limit to asking the people in front of you to settle down (Musician A).

As for the negative aspects, both musicians and teachers experienced technical difficulties that were out of their control (internet connection; problematic issues with the Zoom platform; etc.). In addition, being seated for long periods (working on a computer during the day, then sitting in front of a computer for rehearsal) created some discomfort. Teachers also reported that initially it was challenging to find strategies to engage learners during rehearsals, leading them to a solution whereby musicians were encouraged to unmute and speak whenever they had a question. In terms of motivation, Musician B pointed out that, unsurprisingly, it was more motivating to meet in person than online, but that the latter was better than having nothing at all.

#### Facilitating the Online Transfer

When exploring the impact of young conductors supporting band members through the transfer of in-person to virtual rehearsals, one element that stood out was the musicians’ level of appreciation for their teachers in accompanying them through the experience of making music online. For instance, Musician A shared the following about the teachers’ humanity, as they showed kindness and compassion, used humor and were able to respond to individuals’ needs within the group setting:

I think we really got the best of the best teachers. Not only are they competent, knowledgeable, but they’re also human, kind and honestly, I really enjoyed getting to know [Conductor B] differently than how I saw her when we were face to face. She’s a lot funnier in person and she’s very, very human, really. [Conductor A] is the same. It’s true that we were in a group, but at the same time we were like individuals, so there are human aspects to this that are very valuable to me (Musician A).

Another element reported by both musicians and teachers was the importance of support and feedback, be it through written tutorials or immediate technical help during rehearsals. Conductor B explained: “Most of it was, either one of two things : [1] the tech side of it (making things work so that they can hear and they can participate); And then [2] the technical difficulties of trying to figure out the music on their own.” They added that musicians did not have a recording of their individual parts, which made it difficult for them to isolate their melodic line when playing with the recording and to ensure that they were performing it correctly. Because musicians were not able to receive feedback while playing, conductors later started to provide personalized feedback, which took the form of written comments to musicians who sent individual recordings of themselves playing by email.

In summary, intergenerational relationships were mostly considered positively, especially relating to helping with technological troubleshooting. While a perspective emerged stating that intergenerationality was not a focal point of the learning experience in the virtual environment, participants did not mention negative aspects of having a mixed age range within the group.

### Theme 2: Digital Literacy and Access in Later Life

#### Fostering Digital Literacy and Mitigating the Generational Divide Through Music Technologies

By participating in weekly online rehearsals, musicians explored different technological tools. For instance, the conductors shared a list of online resources that musicians could use in their spare time (e.g., musictheory.net), prepared videos on musical topics for asynchronous learning (e.g., identify major keys and their relative minors), and created various written tutorials using a checklist format (step-by-step guides on how to set-up Zoom and how to record yourself for virtual concerts). As such, the nature of conductor support changed from a sole focus on musical matters to encompassing how to use the technology to mediate the learning. Consequently, we explored whether music technology could allow band members to develop skills that would be transferable to their daily lives and contribute, then, to attenuate the generational digital divide that has been observed by some researchers (Charness and Boot, 2009, p. 256; Poushter, 2016, p. 10). When interviewing the musicians, they perceived that using technology should not necessarily be associated with being young or old, but rather with the level of interest (or fear) one may have:

I think it’s a bit of a stereotype to say that older people are not good in technology, because I’m better at it than quite a few people in my family and other people. So, it more depends on what your interest is and what your familiarity is, and how afraid you are of it. I think a lot of it is overcoming fear (Musician B).

Here, Musicians B described her use of technology in comparison with those of family members, whereas Musician A focused on the fact that levels of expertise did not have to be associated with age: “In life, you deal with all kinds of levels, with people of all ages, and for me, it doesn’t matter at all.” The latter continued by explaining that she would want more intergenerational initiatives but that our society still has a tendency to categorize the population by strata, which results in dividing and polarizing people:

I’ve seen it even more lately, our society divides people. And I would like to encourage activities like those in the Montréal New Horizons Band so that people stop using this age barrier, and stop placing people in packs or classify them. I know that each one of us can learn from someone else (Musician A).

All musicians also commented on the fact that they received a lot of support from their teachers, which contributed to making their experience positive.

We are lucky to have leaders who are competent and generous and kind […]. It’s really comforting to see that there are young people who are interested in that [i.e., intergenerational learning] because we don’t often hear about it, unfortunately (Musician A).

As for the conductors, they reported that to make musicians feel comfortable in the virtual format of the rehearsal required a great deal of preparation on their side. They chose to assume that everybody was novice in using technology and prepared accordingly by creating tutorials and written guidelines:

Making those types of things… not assuming that everyone knows [technology]: Having tutorials, walkthroughs, try to make everything intuitive but not just for people who know how to use a smartphone. Making sure not to rely too much on those assumptions (Conductor B).

In terms of mitigating the generational divide, Conductor A emphasized that because the situation was new to everybody, including the teachers, it contributed to decreasing the digital chasm:

I think it helped close that gap a little bit because, we were navigating it with them too: I had never done that before, teaching them in that way, in an online format, so I think it made them a lot more comfortable, and me too, to be fine with things that didn’t work or if we made mistakes… and even to celebrate the little things that go right (Conductor A).

#### Maximizing Equitable Access in Later-Life

Could virtual rehearsals have the potential to foster equitable access to music? Responses were mixed among participants. For instance, while Musician B indicated that “it really overcomes the barrier of distance and cost for people,” Conductors B and C were more cautious with their answers. Conductor C indicated: “Tricky, because not everyone has the same set up, not everyone has the same access to technology, and that’s been super apparent with what went on at the beginning of the pandemic.” She also added that “using technology requires technology,” which is aligned with Conductor B who indicated that it “requires a lot of investment in the hardware, a good internet connection, and things like that.” That said, Conductor B also mentioned that it made it easier for people to engage in activities: “the online stuff has actually made it in some ways more accessible to people ‘cause it’s less work than to have to go somewhere else to do it, you could just do it from home in your pajamas [laugh].”

In order to maximize access, musicians and conductors indicated that using the Zoom platform to hold the rehearsal was a good choice as it was quite user-friendly and did not require an account for attendees to join. It is worth noting that in addition to the weekly virtual rehearsals, most participants had used that platform on other occasions as well. For instance, Musician B used Zoom to communicate with her family as well as to sing in a choir online: “I belong to this Mandarin choir and we would be meeting on Zoom every other Saturday.”

### Theme 3: Online Music-Making in a COVID-19 Context

The third theme addressed online music-making during the pandemic and members’ perception about their experience. Our first question was: What implications has the shift to an online environment had for you?

From the teacher’s perspective, Conductor C commented on the importance of being able to adapt, to be flexible, to try new things and to be okay if it did not work because at least, you tried. She also emphasized the difference between in-person teaching and online teaching, even for a person as exuberant as herself:

I think it has allowed me to be adaptable of teaching in person and teaching a full classroom—whether it’s a band scenario, whether it’s a classroom of kids for example, vs. lecturing online, it’s made me be able to adapt and flip between two completely different styles of teaching because even though I have an excitable personality and I’m very out there, I actually find it way more exhausting to do that online than to do that in person (Conductor C).

From the musician’s perspective, having access to online rehearsals was definitely better than having nothing at all. Musician B explained that missing rehearsals for a year would have made it very easy to abandon playing music: “I was really glad that you decided to do it online, because missing that year and some… It would’ve been really easy to drop out of it all together, and stop practicing, and then not feel like coming back, and losing touch with all the learnings and the teachers and the other group members.”

Musician A said that because she could continue to do music, she did not feel as if she had given up all aspects of her life to the pandemic: “I didn’t feel like I’d lost everything when the pandemic struck. I would have missed it a lot if I did not have these rehearsals.”

Another element that she (Musician A) and others mentioned was that unfortunately, the shift to online rehearsals caused a drop in the number of members. Musician A indicated that she knew some individuals who did not register because they lived in apartments and could not play their instrument in the evening, out of respect for their neighbors. Others also felt they were just not familiar enough with technology to participate, or simply did not have the equipment to do so.

Teachers and musicians had to develop new skills or resources during their year of doing music online. Conductor A commented on how nice it was to see participants evolve and become more comfortable over time, especially when comparing the very first rehearsal to where they were at the end of the year.

Thinking about where they were in the very first online rehearsal and then seeing them where they are now, is just like a big source of pride. Because I knew it was gonna be hard for them and I knew that we would end up losing people [along] the way because it was gonna be frustrating. [But] seeing them evolve from [thinking], “I don’t really know what we’re doing” to laughing because they can’t turn off the bunny filter [on Zoom], [and also] being able to feel comfortable unmuting to ask a question or to make a joke was really nice (Conductor A).

Additionally, when asked about the pandemic and how music may have helped people go through it, that same conductor told the following story:

[At] the end of our last rehearsal in the spring, [the] cohort seemed to [feel] closer to each other […]. [There] was one point during [Advanced Band] where I had a sectional and they were feeling pretty confident and I felt pretty confident [about] them and we all just checked on each other. [It] had nothing to do with music, we were just asking [each other], “how are you doing? How’s work? How’s school?” “Are you okay?” “What’s going on?” and we just talked for more than half of the sectional, getting to know each other, and that was really really nice ‘cause I would’ve never been able to do that [during in-person rehearsals] (Conductor A).

One may wonder whether this is solely the product of the online environment or whether the circumstances surrounding the pandemic allowed these types of interactions to happen. That said, this quote highlights how social relationships progressively strengthened, suggesting that interpersonal aspects may flourish through online music-making.

Lastly, participants were asked what they felt they would need for a satisfying experience if they were to continue music-making online. From the responses, three requirements were emphasized by both students and teachers: (1) personalized feedback, (2) taking ownership of one’s learning, and (3) support.

#### Personalized Feedback

The majority of rehearsals were held in a sectional style, with musicians separated between instrument families to receive more specific instruction relating to their instruments. Full ensemble rehearsals were held approximately three times per semester. Before these rehearsals, conductors asked musicians to submit individual recordings of themselves performing a run through of the pieces in order to better assess problematic areas and challenges that were not always evident during full ensemble rehearsals (e.g., incorrect rhythms, wrong notes, etc.). Personalized feedback was then sent via email to each musician who submitted a recording, which participants felt was helpful and even an advantage over their previous in-person rehearsals: “Our conductors analyze our recordings, and I think that is so wonderful. That’s an improvement over in-person: you get individual feedback on your performance” (Musician C).

Musician B further underlined the importance of this personalized feedback, saying: “That’s an excellent idea, because otherwise [the teachers don’t] really know if we’re doing anything.” The following anecdote also illustrates how conductors may have been unaware of different issues (technological or otherwise) that could have affected musicians’ ability to follow them:

A couple of times, I couldn’t get the music, it was on my tablet and because of my issues with my technology, I couldn’t get to do it fast enough so I wasn’t even doing it… and [the conductor] didn’t know I wasn’t doing it! [laugh] And one other member was asked to do a little part and I think he wasn’t even looking at the right music at the time… and it didn’t show! [Laugh] If he hadn’t been asked, nobody would’ve known so, I think it puts more of an onus on ourselves to actively participate. But I think most of us are, really, actively participating. I think it’s pretty good considering (Musician B).

While this provided humorous examples of musicians not getting their materials fast enough to play when prompted during ensemble rehearsals, it also meant that conductors were limited in terms of diagnosing the challenges musicians faced when playing together online. This often made it difficult for conductors to gauge what to work on during full band rehearsals.

#### Taking Ownership of One’s Learning

As mentioned by Musician B above, it became the learners’ responsibility to actively participate in order to fully benefit from the virtual music-making experience. This suggests a fundamental change of facilitator style from largely hierarchical to much more cooperative. Taking ownership represented a significant departure from the teaching approach used previously during in-person rehearsals, where conductors tended to take the lead in choosing what to work on based on hearing the ensemble as a whole. Here, in virtual band, conductors indicated that musicians’ ownership of their learning was paramount for lesson planning and rehearsing. For example, they heavily encouraged musicians to ask questions—via Zoom or email—on aspects they were having issues with, or regarding passages they wished to work on more. Conductor B commented on the importance of dialogue:

I think that the engagement, the feedback, and having people talk—having it be more of a conversation—for the people on the other side to take more ownership of that and to come with questions, or to be prepared to stop things and [say]: “I need you to do this, can you explain this again?” (Conductor B)

While some musicians initially felt hesitant to un-mute themselves to ask questions, conductors tried to support the group by saying it was okay to make mistakes, to not know things, and gave resources they may find helpful outside of rehearsal time.

#### Providing Support

Many participants noted that the shift from in-person to online rehearsals was indeed challenging. However, both musicians and conductors supported one another during this process by being open, welcoming, understanding, and by sharing resources.

For example, proper technological set-up (both for equipment and Zoom settings) were required from musicians and conductors. Musician B states: “I think the teachers gave a lot of online resources. And that was helpful. Like how to get online, how to record, and also links to exercises online done by other people. Those were very useful.”

## Discussion

This study addressed three themes: (1) intergenerational relationships to support online group music-making; (2) digital literacy and access in later life; and (3) online music-making in a COVID-19 context. The first theme showed that being part of an intergenerational ensemble composed of younger conductors and older adult musicians contributed positively to the learning experience online. The second theme demonstrated that the generational digital divide reported in research (Charness and Boot, 2009, p. 253; Poushter, 2016, p. 6–23) was not particularly apparent in the context of the Montréal New Horizons Band. In fact, all participants, be they young or old, conductors or musicians, were new to the online format of rehearsals, which contributed to attenuate the age-related differences that may have been observed in other studies. Regarding access in later-life, online rehearsing was beneficial for older musicians in terms of distance/commute, time, energy, and cost. However, for those who did not already have internet and electronic devices, the cost of acquiring all the necessary equipment to make music online could have been too high. Finally, the third theme revealed that musicians appreciated the opportunity to make music online and indicated that it was definitely better than having nothing, especially for its social aspects.

It is worth noting that throughout these themes, several challenges and opportunities were noted by the musicians and conductors.

### Challenges

Overall fatigue was said to be a major challenge for all those involved in the online rehearsals. Musicians and conductors stated that sitting for long periods of time in front of a screen could be especially taxing—particularly since some participants had desk jobs, were already teaching online, or were taking university classes online. As a result, conductors felt they had to “dial up” (Conductor A) their personalities when teaching online, further adding to their overall fatigue due to a perceived need to “fill the void/space” (Conductors A and B) in a virtual environment. Conductors A and B, who were teaching the online bands, both related this to difficulty gauging levels of engagement and the lack of immediate feedback from participants, factors noted in other studies looking at music learning during COVID-19 (Salvador et al., 2021, p. 195; Schiavio et al., 2021, p. 174). Conductor B mentioned feeling the need to talk much more during online rehearsals, which took more energy. This was also paralleled by Conductor C who stated that though she perceived herself to have a more “excitable” personality, it was exhausting to maintain this in an online space. In a paper by Gibson (2021), the author mentions that even those considered to be great educators and mentors may struggle in the virtual environment, as they may feel that talking to a screen is less inspiring than to a room with people (p. 160). Likewise, Thorgersen and Mars (2021, p. 235–237) mention that online teaching can be especially time consuming. Moreover, fatigue often affected musicians’ motivations to practice repertoire, with participants saying that they felt less driven to practice for online rehearsals than for in-person, perhaps due to having to play on mute during rehearsals. This finding is aligned with Akyürek (2020, p. 1804) who also noticed lower motivation to complete homework by undergraduate students in music education in the context of distance learning.

Additionally, online rehearsals require a certain level of equipment, such as a decent internet connection and a functioning computer—things that unfortunately may not be accessible to all people, whether educator or student (Gibson, 2021, p. 159–160; Rowan, 2021, p. 3; Thorgersen and Mars, 2021, p. 226). Oftentimes, the quality of an individual’s technological equipment may serve to propel or hinder the virtual learning experience (Gibson, 2021, p. 161). For example, Schiavio et al. (2021) write that technological tools may play an important part in building “reciprocal presence” among teachers and students, which can help promote student learning and acquisition of skills (p. 172), or create frustrating situations for learners and educators if technological quality is poor (p. 174). Furthermore, scheduling or the availability of home space could get in the way of rehearsals, as participants needed to be mindful of their work hours or their neighbors.

Lastly, the chance that technical issues would emerge was always present in the online format. Unsatisfactory internet connection or equipment simply not working were often sources of frustration for participants and were not always within their control to fix, a finding also reported by Rowan (2021) and Salvador et al. (2021, p. 201). Approaches that were used extensively during in-person rehearsals may not have always translated well into the online environment as well, such as ensemble tuning, isolating parts, or listening for balance.

### Opportunities

Despite these challenges, online music-making also provided participants with several opportunities that perhaps had not been available to them during in-person rehearsals. As was mentioned above, personalized feedback was highly valued by musicians and was an aspect of online rehearsals that many felt was an advantage over in-person rehearsal structure. Though time consuming, both musicians and conductors noted the benefits of this approach, which seemed similar to what one may get from private music instruction.

Surprisingly, many participants had noted that they were able to get to know each other far better online than when they were in-person. Conductors were able to always see participants’ names in the Zoom window, and musicians were not limited to just speaking to their section mates. For example, while clarinets and trombones were situated on opposite sides of the stage during in-person rehearsals, this was a non-issue for virtual rehearsals. This is similar to an observation made by Cheng and Lam (2021), who stated that though student-teacher interactions decreased during the pandemic, when they were able to chat, students appreciated seeing their teachers’ faces and teachers observed more focus or excitement toward learning music (p. 219). Additionally, the virtual environment was a way for individuals to stay in contact with others *and* continue making music, stating that it was beneficial to their lives during the pandemic.

Similar to the way that physical proximity within the group could inhibit social contact within place-based rehearsal but ceased to be a barrier within the online environment, geographic location, weather, and instrument weight were no longer issues. Musicians could freely attend rehearsal regardless of extreme weather or public transit malfunction—both of which Montréal is well-known. Prior to the pandemic, in-person rehearsals were held at a building located near the top of a steep hill. For those playing larger instruments like tuba or tenor sax, the transport of their instruments or walking up a potentially icy hill during wintertime was no longer a barrier for participation. These convenient factors were also noted by Salvador et al. (2021), who stated that no commuting, being comfortable at home, and not carrying heavy equipment were appreciated by young and adult students, as well as parents of students (p. 199). Additionally, some participants had expressed interest in picking up new instruments for the intermediate band while remaining on their main instrument for the advanced band. Virtual rehearsals made it easier for them to switch between instruments without having to worry about transporting two instruments or having their cases being cumbersome for fellow bandmates to sit next to. It also made it easier to see the conductor when playing. Interestingly, the online environment played a role in mitigating some “structural barriers” associated with active music-making that have been identified by Creech and colleagues (2014, p. 138), such as location and transport issues. They also identified finance as a structural issue, which, in this case, was taken into account by offering the virtual band at half the price of the regular in-person activities.

Lastly, some musicians had mentioned they were more able to move at their own pace. Rehearsals and sectionals were recorded and made available to ensemble members, providing them a way to catch up if they were absent during a rehearsal, or to simply go back and watch segments whenever they wished. This showcases the potential for online music-making in helping to support an individual’s own practice (Gibson, 2021, p. 153).

### Virtual Rehearsals: Some Ambiguities

There were some aspects of virtual rehearsals, however, that did not entirely fit under a challenge or opportunity, and instead provided both. Perhaps the most obvious change between online and in-person rehearsals was the ability to play synchronously while hearing one’s peers and experiencing the sense of being part of an ensemble. While virtual rehearsing did not lend itself well to exploring issues such as group intonation or balance, many musicians felt more at ease when playing because no one but themselves could hear their mistakes. Additionally, though conductors admitted that it was challenging to learn to adopt a more flexible or adaptive teaching style, it was also a valuable learning experience. Conductors noted that they were able to think “on their feet” better (Conductor A and C) and were more open to creative solutions and approaches in teaching. Lastly, while asking musicians to take more ownership over their learning could be a challenge, it was also an opportunity for them to practice critical self-reflection on what they did well and what they needed to improve. Some musicians become noticeably more comfortable over time pausing rehearsals to ask questions or clarify things with which they were uncertain.

Despite the ease with which we dichotomize in-person and online learning, the ambiguities listed above may help to emphasize that each approach has pros and cons that should be considered when building music learning environments, particularly in a post-pandemic world (Camlin and Lisboa, 2021, p. 137).

### Pedagogical Implications

From these findings, we propose the following pedagogical implications for future online music environments.

#### Support Network

Many interviewees noted that they were able to get to know other members better online than even in-person. Here, the online music environment provided a support network for those involved, no matter their age. Musicians were not only able to learn more about their bandmates, but they were able to get to know the conductors better as well—reducing the “space,” both physical and psychological, between musician and conductor, and allowing meaningful intergenerational relations. This support has been echoed in other papers examining musical activities through the pandemic. In an editorial by Camlin and Lisboa (2021), the authors note a common theme among their received papers, stating that though there existed several disruptions, many people turned toward each other for “practical, psychological, familiar, peer, and/or collegiate support” (p. 135). Additionally, online music helped members stay in contact with each other despite the pandemic’s social distancing rules. Members were able to have a weekly event where they could share interests, chat, and check in on each other. This contributed to the overall “welcoming” feeling of the online music ensemble, with one musician stating that perhaps being welcomed was something everyone needed during the pandemic. Schiavio et al. (2021) note that the shift to online may have helped people realize how important having a community was to them particularly when experiencing challenges, providing a source of encouragement and advice (p. 173). This also aligns with Thornton’s (2020) reflection about how, in response to the pandemic, a stronger sense of community was developed among music teachers and artists.

While providing a welcoming space was also emphasized during the Montreal New Horizons Band’s in-person rehearsals before the pandemic (through common coffee breaks, for instance), most people tended to speak with musicians they already knew and to defer to the conductors to make most decisions, which created, although involuntarily, some form of hierarchy. Because the online environment took away that physical space factor that can so often reinforce power relationships, it placed people on a level playing field as all, musicians and conductors alike, were new to virtual music-making. There is thus a strong case to be made for in-person ensembles to consider encouraging a support network by incorporating some kinds of online elements (for instance, online meet-and-greets or get-togethers, virtual jams) to reproduce this sense of community with equal status.

#### Meet People Where They Are

Providing a welcoming environment, aligned with the “meet people where they are” mentality the conductors adopted, meant that they made no assumptions about anyone’s ability with technology. For example, though the young conductors may have been more accustomed to using technology in their daily lives, they did not assume that their older amateur musicians had the same level of comfort they did. In an effort to improve technological fluency and to help band members to feel at ease, a set-up checklist with instructions on how to position one’s computer, music stand, and instructions for Zoom settings was provided to all musicians. This is in line with a conclusion from Vaportzis et al. (2017, p. 1), who state that providing help in the earlier learning process in order to facilitate the adoption of technology for independent living was just as important as understanding older adults’ perceptions on using technology.

Conductors underlined that it was fine to make mistakes—especially as not everything in an online environment could be controlled. Often, even conductors experienced internet problems, bugs, or technological mistakes that the whole ensemble could laugh about together, while also teaching them to be flexible in an online teaching environment. In his experience with the New Horizons Band Guelph, Rowan also commented on the fact that “each trial presented a different challenge” and that his capacity to “adapt to technical troubleshooting” became his “most important skill as a facilitator” (2021, p. 2). Conductors and teachers should consider providing a safe space to interact with technology, as it has been suggested to encourage technology adoption among older adults (Heinz, 2013, p. 69–70). Importantly, it has been mentioned that while learners need support and resources in order to benefit from the online environment, that educators must also feel well-supported in their endeavors to ensure that the teaching experience they give is not adversely compromised (Camlin and Lisboa, 2021, p. 137). This highlights the importance of reciprocal support between both educator and learner (Gibson, 2021, p. 154), particularly as how, when the pandemic began, many music educators felt they were not ready to make the “shift,” which in turn affected their wellbeing (Cheng and Lam, 2021, p. 211). As mentioned above, organizing online events could be a solution to facilitate access and increase digital literacy while providing some form of social support.

#### Learning to Adapt

Teaching online was often found to be more exhausting for the conductors compared to in-person teaching, perhaps due to more of a “lecture-style” approach. Because of this, conductors heavily encouraged musicians to be more proactive in what and how they wanted to learn, reflect honestly on what they did well or needed to work on, and ask questions during the rehearsal or via email. In some cases, raising one’s hand in an in-person classroom setting may have been easier than unmuting on Zoom, but interestingly, Musician A noted that they felt themselves grow braver in asking questions because of their online experience.

Musicians unanimously stated that the idea of personalized feedback was an advantage to online music, especially as it was difficult to play synchronously and therefore not always possible for the conductor to gauge problematic areas in pieces. It is important to note that musicians perceived this as an advantage over in-person rehearsals, as they would not have necessarily received this level of personalization unless they were taking private lessons on the side. This approach not only helped the musicians but also the conductors in noticing common mistakes among the recordings, signaling to them that this was an area to work on during full band rehearsals. However, it is worth noting that this approach may not be sustainable in the long-term as it was time-consuming for teachers and may not have been, in fact, what group music-making really aims to achieve. This perhaps is a real indicator of the limitations of the online environment—i.e., that the context may have propelled conductors toward using personalized feedback and encouraged participants to seek it, but in truth, it was not a traditional form of group music-making. Even if conductors could be properly resourced to do this, personalized feedback does not replace the situated peer learning and support that can be achieved in an in-person environment.

Additionally, during in-person rehearsals, the conductor was often in charge of lesson planning based on past rehearsals. However, the online music environment pushed conductors to be flexible in their teaching approach, using pre-planned content, adapting to the needs of the learners, improvising, trying new methods, or any combination of these. Being responsive to students’ needs is a characteristic that has been identified by Thornton (2020) as a consequence of the pandemic. Moreover, while in-person rehearsals had the conductor for the most part choosing what to work on, online music teaching became more of a team effort between teachers and musicians. For instance, the idea of trying to play chorale-like pieces unmuted was something both musicians and teachers decided to explore together. This democratic approach should be worth replicating, even with in-person teaching, as it contributes to musicians’ ownership of learning, a key component of musical engagement (Creech et al., 2014). In addition, a willingness among music teachers to creatively adapt and be flexible may also reflect an openness to transition from being instructors to becoming facilitators of their students’ learning, which could promote the latter’s autonomy, even beyond the pandemic crisis (Camlin and Lisboa, 2021, p. 137).

#### Collaborative Teaching

For this online ensemble, teaching became less of a solo endeavor and more of a team effort in order to find new and effective ways of delivering quality musical experiences to the ensemble. Even during full band rehearsals, where one conductor took the lead, the other often stayed on in the background to provide technological support, answering the Zoom chat, helping the main conductor answer questions, etc. Conductors often met in their free time to discuss their approaches—what they felt worked, didn’t work, solutions, and other areas of note.

As a humorous example, Conductors A and B recalled an instance where Conductor B’s (the main conductor for this particular session) internet was not stable enough for video, and therefore she couldn’t conduct. Both conductors ended up working together so that Conductor B would play audio and give instructions to the ensemble, while Conductor A directed on video. Though both conductors were initially convinced this approach would fail, it ended up being surprisingly successful. The evidence seems to suggest that team teaching should be strongly encouraged in the context of online music-making, to reduce the cognitive load of the conductors as one can focus on the music and the other on technological issues.

### Limitations

Some limitations must be acknowledged. The aim of our study was to document a situation where music practices were transformed to adapt to the constraint of the pandemic and to report it through first hand experience of participants. In that sense, this study does not have results that are generalizable. While our sample was made of culturally diverse individuals, they all came from higher socio-economic backgrounds, meaning that they had the financial means to access technology. As such, we missed the voices of those with lower incomes, who could have provided a different perspective about equitable access. All participants who agreed to be interviewed for this study were already familiar with technology, which may not be representative of all musicians within the Montreal New Horizons Band. Furthermore, they already knew how to play their instrument. During the months that this project took place, no novices were offered the opportunity to start learning an instrument online with the band, because it was deemed too complex to provide an introduction to music on multiple instruments to a group of beginners online. Therefore, novice musicians were outside our study parameters.

Another limitation of the current study is that participants only included those who stayed active within the ensemble during the pandemic (i.e., less than 30 musicians out of 86). This means that those who did not participate or those who did not participate throughout the three semesters (fall 2020, winter and spring 2021) may have viewed certain challenges and opportunities differently than those who stayed consistently active throughout the pandemic. For example, musicians in the Allegro band, who were more comfortable on their instruments, seemed to have been more likely to continue participating in online activities for two or three semesters, whereas fewer musicians in the Adagio band continued their participation in two or three consecutive semesters. This could perhaps be due to advanced band members being more comfortable on their instruments, while intermediate band members may have had to deal with more musical challenges in addition to the technological ones. This could also be due to the fact that they may not have been part of the MNHB for as long as the more advanced musicians, which means that their sense of belonging may have been weaker. Another hypothesis is that the more advanced musicians had experienced the benefits of in-person music-making for longer, so they may have been more motivated to persevere until the situation could come back to normal.

In terms of study design, we chose to complete interviews after 9 months of participation rather than do pre/post interviews, which would have allowed us to explore how perceptions evolved over time. This choice originated from the fact that one of the authors was also one the conductors of the virtual bands. At the time, she was not aware that the study would take place, so she focused fully on her conductor role before joining the research team to analyze results.

## Conclusion

“*Among all the conversations about logistics, there has been a constant call for empathy, patience, and thoughtfulness for student needs. Educators were leading voices in reminding society that we have to think about everyone as in need of care.*” (Thornton, 2020, p. 4)

The aim of this paper was to examine the effects of online group music-making on community band conductors and older amateur musicians who had to stop in-person rehearsals because of COVID-19. Intergenerational relationships were explored, as well as participants’ perceptions about digital literacy, access in later life, and online music-making in a COVID-19 context. Risks and opportunities were discussed, and results showed that despite the multiple technological and musical challenges reported, participating in online musical activities also offered new opportunities, like a greater flexibility in the learning experience and a renewed sense of community among band members.

The pandemic has been an incredibly trying situation, with many of us struggling with feelings of increased isolation and anxiety. Group music making—which many use as a source of wellbeing and comfort—faced challenges from the shift of traditionally in-person rehearsals to virtual rehearsals. COVID-19 also had an impact on quality of life, and older adults had to find creative avenues to sustain wellbeing during this period. For musicians, it meant having to engage with technology to pursue their artistic goals and to continue benefiting from the social aspect of collaborative music-making. In the end, participants were grateful to have had the opportunity to keep music in their lives while everything else had stopped.

Ultimately, this situation has allowed teachers and students to explore and identify strategies that, not only were effective in the pandemic context, but will also be transferable to the post-pandemic musical context. While the use of online tools or technology for music participation and education was in use pre-pandemic, the actions of musicians, students, and educators have showcased their creativity and resilience in learning to adapt and innovate to these emerging challenges and opportunities.

## Data Availability Statement

The datasets presented in this article are not readily available because some information contains identifiable data. Requests to access the datasets should be directed to A-KB, barbeau.audrey_kristel@uqam.ca.

## Ethics Statement

The studies involving human participants were reviewed and approved by the Comité Institutionnel d’Éthique de la Recherche avec des Êtres Humains de l’Université du Québec à Montréal. The patients/participants provided their written informed consent to participate in this study.

## Author Contributions

A-KB designed the research project, collected the data, and wrote the article. MG contributed to the data analysis, the redaction of the article, and the formatting of the manuscript. AC contributed to the review of the literature, acted as an adviser for qualitative analyses, and contributed to the review of the article. All authors contributed to the article and approved the submitted version.

## Conflict of Interest

The authors declare that the research was conducted in the absence of any commercial or financial relationships that could be construed as a potential conflict of interest.

## Publisher’s Note

All claims expressed in this article are solely those of the authors and do not necessarily represent those of their affiliated organizations, or those of the publisher, the editors and the reviewers. Any product that may be evaluated in this article, or claim that may be made by its manufacturer, is not guaranteed or endorsed by the publisher.

## Footnotes

1 Arrangements that divided musical parts into “voices,” similar to soprano-alto-tenor-bass (SATB) choir music, instead of via instrument. Flex band pieces typically ranged from 4 to 6 parts with percussion/keyboards or auxiliary instruments.
