# Supplementary material for: Online Group Music-Making in Community Concert Bands: Perspectives From Conductors and Older Amateur Musicians
Source: Front Psychol. 2022 Jun 30;13:878307. doi: 10.3389/fpsyg.2022.878307 (PMC9285893; doi:10.3389/fpsyg.2022.878307)
Supplement: Supplementary file 1 [file Presentation_1.pdf]

Additional File

A Leap of Faith: Online Music-Making in Community Concert Bands

Authors:
Audrey-Kristel Barbeau, PhD
Département de musique, Faculté des arts
Université du Québec à Montréal

Mariane Generale, PhD candidate
Schulich School of Music
McGill University

Andrea Creech, PhD
Schulich School of Music
McGill University

Appendix I: Structure of MNHB online rehearsals

- A majority of the rehearsals were held as sectional format, with musicians being split into their instrument families (woodwinds; brass and percussion)
- Full band rehearsals were held three times a semester (beginning, middle, end)
- All participants, except the conductor, were asked to have their microphones muted unless called on by the conductor. Musicians were encouraged to use the "raise hand" function or interrupt the rehearsal by unmuting if they had a question
- Musicians played along with the conductor on keyboards or with the conductor directing to a recording of the piece
    - Recordings were manipulated to be slower tempi using AudioTrimmer
    - For slower, chorale-like pieces and warm-ups, it was possible to have everyone unmute and play together
- Duration:
    - Fall 2020: 1 hour per group
    - Winter 2021: 1 hour 15 minutes per group
        - Zoom rooms were open before the first rehearsal; a 15 minute break was added between the two groups to encourage socialization
- Both bands ended their semesters by individually recording themselves playing a selected piece to be put together for a "virtual concert" video.

During the first rehearsal of every semester, much time was spent ensuring every musician had the proper Zoom settings. Rehearsals ran as follows:

- Conductors began with announcements if applicable, or answered any questions musicians had
- A brief warm-up was done with the full band, even on scheduled sectionals. Sometimes music theory or music history lessons were also done during this time
- The ensemble would separate for sectionals into break-out rooms or stay together during full band rehearsals to work on pieces
- The rehearsal would end with a run-through of a piece (time permitting) or a question period

All rehearsals and sectionals were recorded and made available to ensemble members during the semester. Permission was asked at the beginning of each semester if participants were comfortable showing their faces in these recordings. Participants who indicated no were asked to keep their camera off during rehearsals.

The following table shows MNHB's rehearsal schedule for Fall of 2020, Winter and Spring of 2021.

Fall 2020 (60 min)		Winter 2021 (75 minutes)		Spring 2021 (75 minutes)	
Week	Type	Week	Type	Week	Type
1	Full band	1	Full band	1	Full band
2 to 7	Sectionals	2 to 7	Sectionals	2 and 3	Sectionals
8	Full band	8	Full band	4	Full band
9 to 11	Sectionals	9 to 11	Sectionals	5 to 7	Sectionals
12	Full band	12	Full band	8	Full band

Appendix 2 : Interview questions

**Theme 1: Intergenerational relationships to support online group music-making**
**1- Teacher:** You taught a virtual ensemble that was intergenerational. How do you think this intergenerational aspect helped musicians in your group? Can you provide examples of intergenerational learning or collaborations?<br><br>**1- Musician:** You participated in a virtual ensemble that was intergenerational. Do you feel that this intergenerational context may have improved your learning experience? If so, how?<br><br>**2- Both:**<br>If you think about your virtual music making experience with the MNHB, what worked particularly well and why, in your opinion?<br><br>**3-Both:**<br>What did not work and how could it be improved?<br><br>**4-Both:**<br>If you had to continue with an online format in the future for your musical practice, what would you absolutely need for it to be satisfying? (Best practice: prompt for concrete examples and naming resources that would be used)<br><br>**5-As a teacher**, how did you support band members in the transfer from face-to-face to virtual rehearsals?<br><br>**5-As a musician,** how helpful was your teacher in supporting the transfer from face-to-face to virtual rehearsals? Did you receive help from other musicians (and if so, how exactly)? Do you consider this to be intergenerational learning (please explain)?
**Theme 2: Digital literacy and access in later-life**
**Both:**<br>**6-**(*Context: There exists a well-documented generational divide regarding the use of technology, with older adults being less likely to engage with technology than what is observed among younger generations (Charness and Boot 2009; Poushter 2016).*) In what ways might emerging music technologies foster digital literacy and mitigate the generational digital divide in Canada and elsewhere?<br><br>**7-**How can virtual music-making maximize equitable access in later-life to creative musical expression, engagement in learning, digital literacy and quality of life amongst older citizens?

**Theme 3: Online music making in a Covid-19 context**
**8-**What implications has the shift to an online environment had for you as a musician (or teacher)?<br><br>**9-**What new opportunities do you think have arisen from the pandemic and having to transfer rehearsals to a virtual format? And for you personally, what did it bring?<br><br>**10-**What challenges have arisen from the pandemic and how have those challenges been addressed or resolved?<br><br>**11-**What new skills or resources have you had to develop, in response to opportunities or challenges raised by the pandemic?<br><br>**12-** We are still living with the pandemic. How is it for you? Does music help you go through it?<br><br>**13-**Any other comment you would like to add?
